# Supplementary material for: MicroPET Imaging Assessment of Brain Tau and Amyloid Deposition in 6 × Tg Alzheimer’s Disease Model Mice
Source: Int J Mol Sci. 2022 May 14;23(10):5485. doi: 10.3390/ijms23105485 (PMC9146140; doi:10.3390/ijms23105485)
Supplement: Supplementary file 1 [file ijms-23-05485-s001.zip › ijms-1681728-SI.pdf]

MicroPET imaging assessment of brain tau and amyloid deposition in 6xTg Alzheimer's disease model mice

ShinWoo Kang[1,2,5], Jinho Kim[2, 3], Sang-Yoon Lee [2, 3, 4], Keun-A Chang[1,2,3*]

[1] Department of Pharmacology, College of Medicine, Gachon University, Incheon 21999, Korea; Kang.Shinwoo@mayo.edu

[2] Neuroscience Research Institute, Gachon University, Incheon 21565, Korea; jinho.k.0331@gmail.com

[3] Gachon Advanced Institute for Health Science and Technology, Graduate School, Gachon University, Incheon 21999, Korea; keuna705@gachon.ac.kr

[4] Department of Neuroscience, College of Medicine, Gachon University, Incheon 21936, Korea; rchemist@gachon.ac.kr

[5] Department of Molecular Pharmacology and Experimental Therapeutics, Mayo Clinic, Rochester, Minnesota 55905, USA

* Correspondence: keuna705@gachon.ac.kr; Tel.: +82-32-899-6411

Supplementary figures

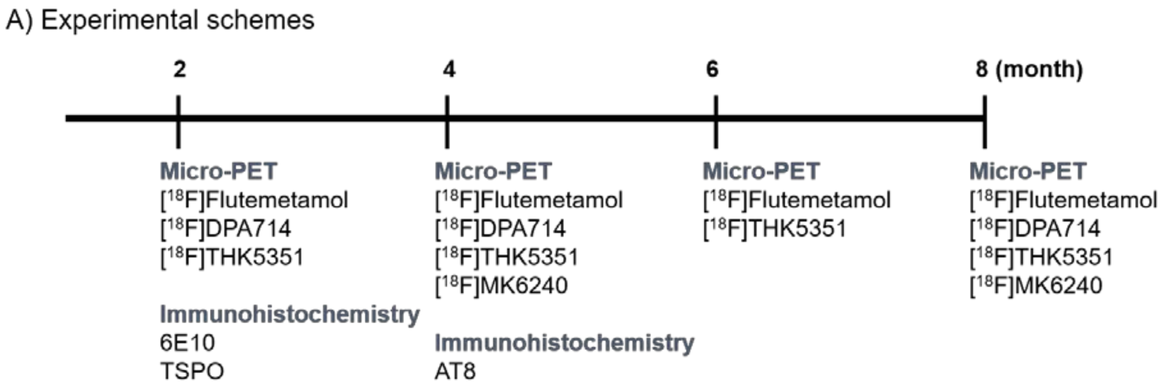

Supplementary Figure 1. Experimental scheme of microPET and immunohistochemistry.

6xTg mice and age-matched WT, JNPL3, or 5xFAD littermates were used for microPET analysis using [^{18}F]Flutemetamol, [^{18}F]DPA714, [^{18}F]THK5351, or [^{18}F]MK6240 and immunohistochemical analysis with 6E10, TSPO or AT8 antibodies.

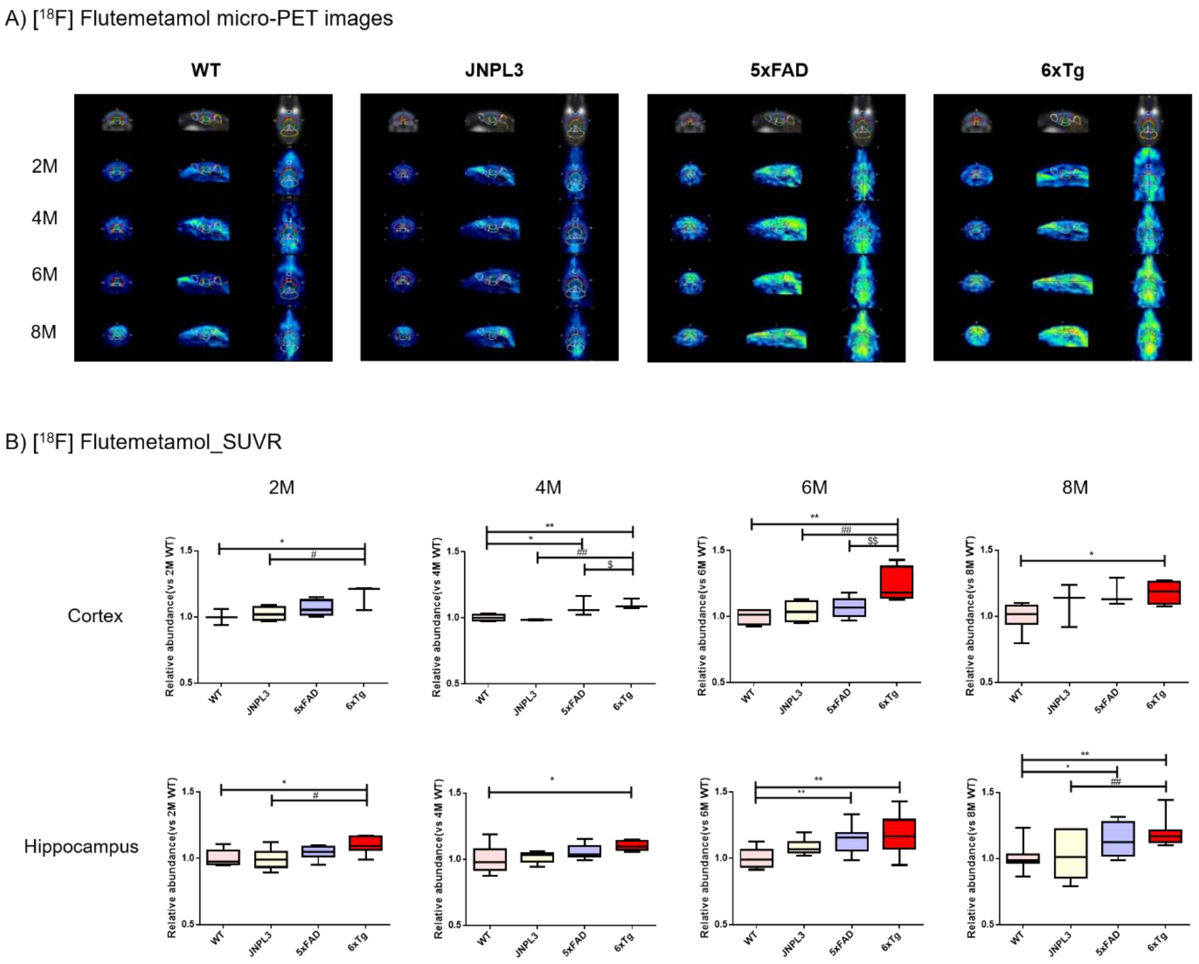

Supplementary Figure 2. Age-associated [18F]Flutemetamol micro-PET imaging in the brains.

2-, 4-, 6- and 8-month-old 6xTg mice and their age-matched WT, JNPL3, or 5xFAD littermates were used for analysis (n=3~6 per group). (A) Representative [18F]Flutemetamol micro-PET images in WT, JNPL3, 5xFAD, and 6xTg mice. (B) ROI-to-cerebellum ratios from 18F-radioactivity data measured over 50 to 60 min were calculated for cortex or hippocampus region in the brains of WT, JNPL3, 5xFAD, or 6xTg mice. All data are given as means ± SEM. The statistical analyses were performed by one-way ANOVA followed by the Fisher exact test. $^{**}p<0.01$, $^{*}p<0.05$ vs WT, ##$p<0.01$ vs JNPL3, $$$p<0.01$, $$p<0.05$ vs 5xFAD.

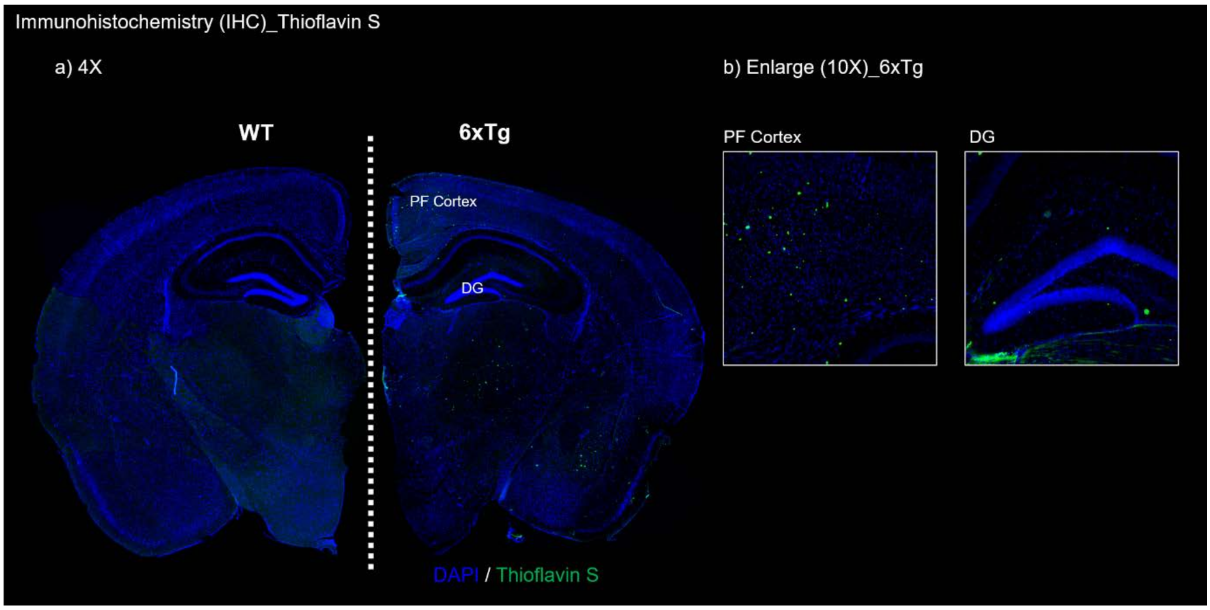

Supplementary Figure 3. Representative images of Thioflavin S stained image in brain.

Brain tissues of 2-month-old 6xTg and WT mice brains were stained with Thioflavin S and counterstained with DAPI. Representative slices are shown for (a) WT and 6xTg mice brain (4x magnification) and (b) cortex (PF) and hippocampus (DG) region of 6xTg mice brain (10x magnification) (all scale bars=100um). Thioflavin S stained Aβ deposits are shown in the cortex and in the hippocampus of 6xTg mice brain.

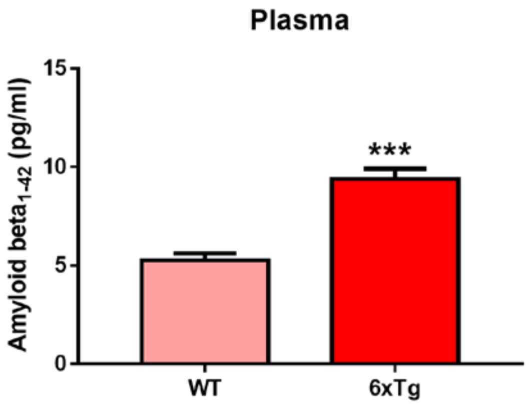

Supplementary Figure 4. The protein levels of Aβ_{1-42} in the blood plasma of mice.

The protein level of Aβ_{1-42} was analyzed in the blood plasma of 2-month-old 6xTg mice and their age-matched WT littermates were used for analysis (n=4~6) per group). All data are given as means ± SEM. The statistical analyses were performed by one-way ANOVA followed by the Fisher exact test. ***$p<0.001$ vs WT.

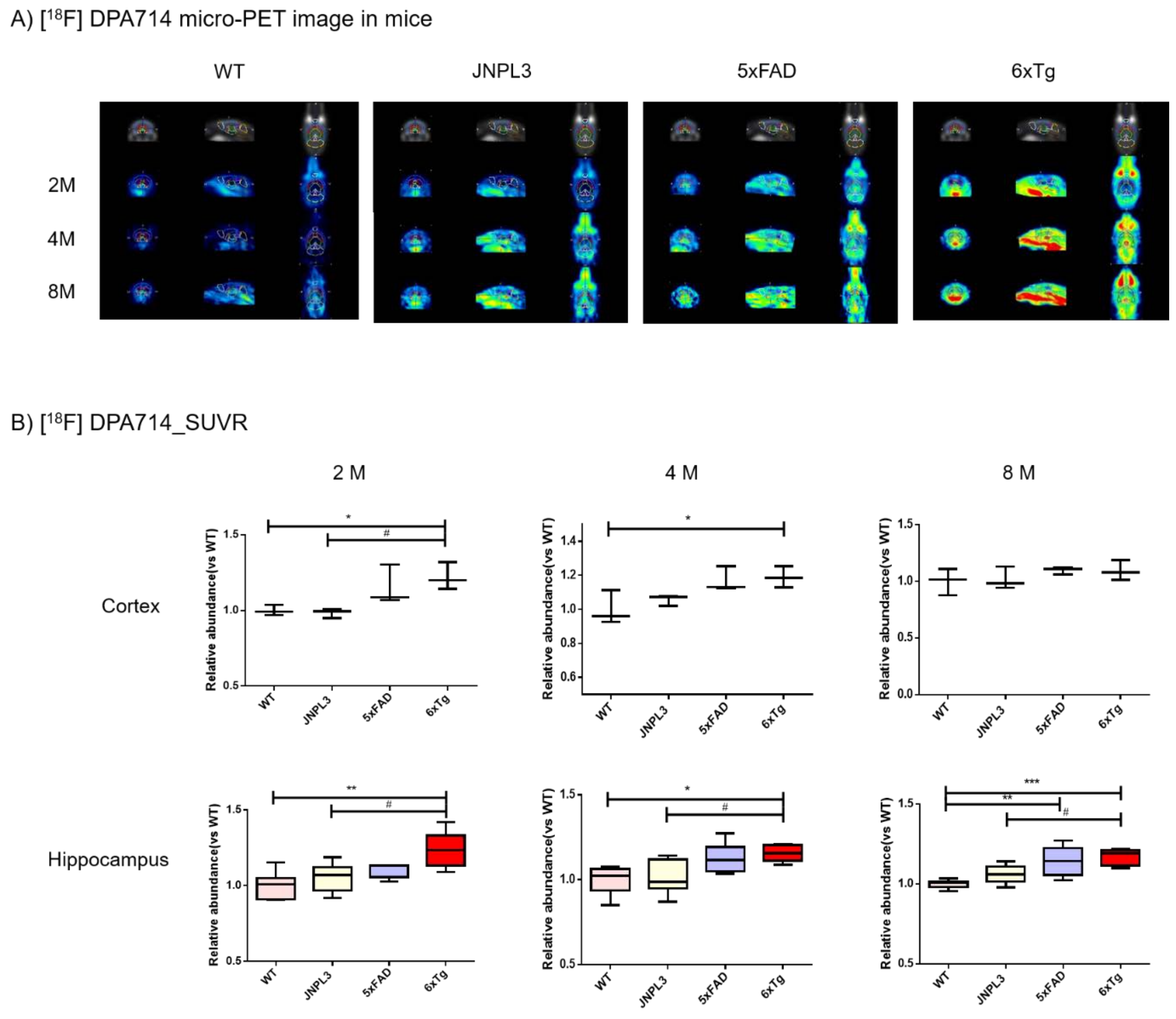

Supplementary Figure 5. Age-associated [^{18}F]DPA714 micro-PET imaging in the brains.

2-, 4-, and 8-month-old 6xTg mice and their age-matched WT, JNPL3, or 5xFAD littermates were used for analysis (n=3~6 per group). (A) Representative [^{18}F]DPA714 micro-PET images in WT, JNPL3, 5xFAD, and 6xTg mice. (B) ROI-to-cerebellum ratios from ^{18}F-radioactivity data measured over 50 to 60 min were calculated for the cortex or hippocampus region in the brains of WT, JNPL3, 5xFAD, or 6xTg mice. All data are given as means ± SEM. The statistical analyses were performed by one-way ANOVA followed by the Fisher exact test. **p<0.01, *p<0.05 vs WT, ##p<0.01 vs JNPL3, $$$p$<0.01, $$p$<0.05 vs 5xFAD.

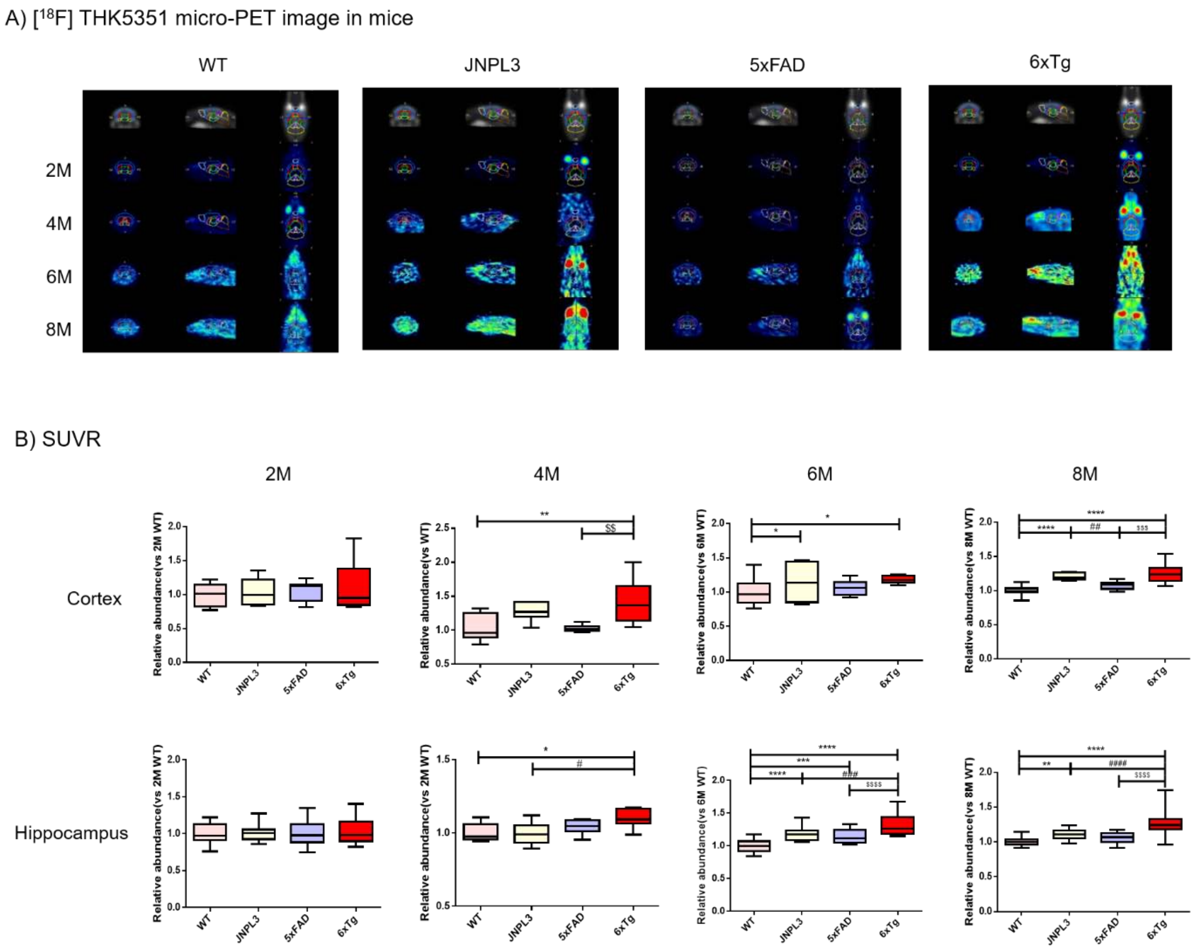

Supplementary Figure 6. **Age-associated [18F]THK5351 micro-PET imaging in the brains.**

2-, 4-, 6-, and 8-month-old 6xTg mice and their age-matched WT, JNPL3 or 5xFAD littermates were used for analysis (n=3~7 per group). (A) Representative [18F]THK5351 micro-PET images in WT, JNPL3, 5xFAD and 6xTg mice. (B) ROI-to-cerebellum ratios from ^{18}F-radioactivity data measured over 50 to 60 min were calculated for cortex or hippocampus region in the brains of WT, JNPL3, 5xFAD or 6xTg mice. All data are given as means ± SEM. The statistical analyses were performed by one-way ANOVA followed by the Fisher exact test. ****p<0.0001, ***p<0.001, **p<0.01, *p<0.05 vs WT, ####p<0.0001, ###p<0.001, ##p<0.01 vs JNPL3, $$$$$p$<0.0001, $$$$p$<0.001, $$$p$<0.01, $$p$<0.05 vs 5xFAD.

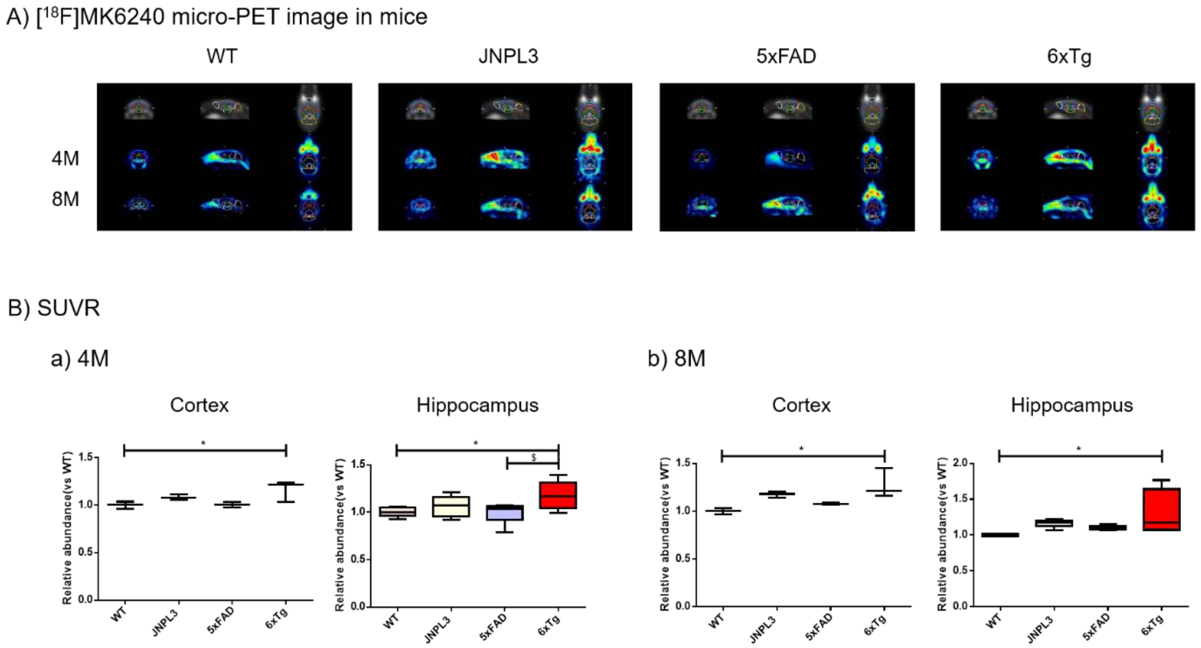

Supplementary Figure 7. Representative images of [18F]MK6240 micro-PET imaging in the brains.

4-, and 8-month-old 6xTg mice and their age-matched WT, JNPL3 or 5xFAD littermates were used for analysis (n=3~7 per group). (A) Representative [^{18}F]MK6240 micro-PET images in WT, JNPL3, 5xFAD and 6xTg mice. (B) ROI-to-cerebellum ratios from ^{18}F-radioactivity data measured over 50 to 60 min were calculated for cortex or hippocampus region in the brains of WT, JNPL3, 5xFAD or 6xTg mice. All data are given as means ± SEM. The statistical analyses were performed by one-way ANOVA followed by the Fisher exact test. ****p<0.0001, ***p<0.001, **p<0.01, *p<0.05 vs WT, ####p<0.0001, ###p<0.001, ##p<0.01 vs JNPL3, $$$$$p$<0.0001, $$$$p$<0.001, $$$p$<0.01, $$p$<0.05 vs 5xFAD.
